# Flow physics and modeling in extreme ultraviolet sources

**DOI:** 10.1093/nsr/nwag298

**Published:** 2026-05-22

**Authors:** Kai Leong Chong, Bo-Fu Wang, Feng Wang, Rui Yan, Hang Ding, Chao Sun, Quan Zhou

**Affiliations:** Shanghai Key Laboratory of Mechanics in Energy Engineering, Shanghai Institute of Applied Mathematics and Mechanics, School of Mechanics and Engineering Science, Shanghai University, Shanghai 200072, China; Shanghai Key Laboratory of Mechanics in Energy Engineering, Shanghai Institute of Applied Mathematics and Mechanics, School of Mechanics and Engineering Science, Shanghai University, Shanghai 200072, China; New Cornerstone Science Laboratory, Center for Combustion Energy, Key Laboratory for Thermal Science and Power Engineering of Ministry of Education, Department of Energy and Power Engineering, Tsinghua University, Beijing 100084, China; Department of Modern Mechanics, University of Science and Technology of China, Hefei 230027, China; Department of Modern Mechanics, University of Science and Technology of China, Hefei 230027, China; New Cornerstone Science Laboratory, Center for Combustion Energy, Key Laboratory for Thermal Science and Power Engineering of Ministry of Education, Department of Energy and Power Engineering, Tsinghua University, Beijing 100084, China; Shanghai Key Laboratory of Mechanics in Energy Engineering, Shanghai Institute of Applied Mathematics and Mechanics, School of Mechanics and Engineering Science, Shanghai University, Shanghai 200072, China

**Keywords:** extreme ultraviolet source, laser-produced plasma, laser-droplet interaction, debris mitigation

## Abstract

The development of high-power extreme ultraviolet (EUV) light sources based on the laser-produced plasma (LPP) principle is crucial for advanced lithography, enabling the continued scaling of semiconductor devices. However, the underlying processes—involving laser-droplet interaction, plasma evolution, and debris transport—are governed by complex, multiscale, and multiphysics phenomena that pose significant challenges for both simulations and experiments. This review comprehensively surveys recent advances in the flow physics and modeling of LPP-EUV sources, focusing on three key areas: (i) numerical modeling and simulation of laser-tin droplet interaction, including laser absorption, radiation transport, equation of states, etc.; (ii) progress in understanding droplet dynamics under femtosecond to nanosecond laser pulses; and (iii) the flow physics of debris transport and mitigation strategies such as buffer gas jets and magnetic fields. By integrating insights from simulations, experiments, and theoretical analyses, this review aims to bridge the gap between fundamental flow physics and applied EUV source engineering, providing a valuable reference for researchers working toward more efficient and debris-resistant EUV light sources.

## Teaser

This review connects fluid dynamics in laser-droplet interactions with EUV engineering, providing a roadmap for more efficient, stable, and debris-resistant advanced semiconductor manufacturing systems.

## INTRODUCTION

Advanced lithography stands as a cornerstone of the semiconductor and integrated circuit industries, enabling the continuous miniaturization and performance enhancement of electronic devices. Among the various lithographic technologies, extreme ultraviolet (EUV) lithography has emerged as the leading solution for manufacturing sub-10 nm node chips, owing to its ability to achieve unprecedented resolution using 13.5 nm wavelength light [1,2]. The successful deployment of EUV lithography into high-volume manufacturing represents a landmark achievement in both optical engineering and materials science.

A critical subsystem of an EUV lithography machine is the light source, requiring high-power, stable, and spectrally pure EUV radiation. The current industry-standard EUV source is based on the laser-produced plasma (LPP) principle [1–5]. In LPP, a high-power CO$_2$ laser is used to irradiate micrometer-sized tin droplets in a low-pressure hydrogen environment. The laser energy transforms the tin into a high-temperature plasma, which emits strongly at 13.5 nm. This EUV light is then collected by specialized mirrors, spectrally filtered, and directed into the lithographic scanner for wafer exposure. However, this process is plagued by two major challenges: low energy conversion efficiency (CE) and severe debris contamination. For instance, ASML’s state-of-the-art Twinscan EXE:5000E lithography system employs a CO$_2$ drive laser with a power exceeding 40 kW to deliver 500 W of usable EUV power at the intermediate focus (IF). More critically, the expansion and cooling of the tin plasma generate significant debris, including energetic ions, weakly charged particles, and micro-droplets, which can deposit onto the collector optics.

The underlying physics of EUV generation via LPP involves multiple scientific and technical challenges. First, the process spans extreme temporal and spatial scales: from laser-droplet interactions and plasma dynamics (nanoseconds and micrometers) to debris formation (microseconds to milliseconds, centimeters), and finally to debris transport and deposition (seconds and meters). This range covers nine orders of magnitude in time and six in length, posing formidable demands on modeling and simulation. Second, the problem involves strongly coupled multi-physics phenomena, including droplet hydrodynamics, laser-plasma interactions, plasma radiation, non-equilibrium thermodynamics, and fluid-particle interactions. Third, experimental observation and data acquisition are extremely difficult, particularly during the initial stages of plasma formation and debris generation, where high-resolution measurements are lacking, thereby limiting model validation and refinement.

As the development of LPP-EUV sources has progressed, several outstanding and highly influential review articles have been published, comprehensively summarizing the physical mechanisms of EUV emission. For instance, the seminal works by O’Sullivan *et al.* [6], Banine *et al.* [7], and the comprehensive overview by Versolato [8] heavily focus on the atomic physics of highly charged tin ions, non-local thermodynamic equilibrium (non-LTE) population kinetics, EUV spectroscopy, and the optimization of radiation conversion efficiency (CE). While the atomic and plasma radiation physics of the main pulse interaction are undeniably the core of EUV generation, the entire LPP-EUV process—from target delivery to collector protection—is fundamentally governed by highly transient, multiscale fluid dynamics and multiphase flow physics. To date, a comprehensive review exclusively dedicated to these hydrodynamic phenomena and their corresponding computational fluid dynamics modeling strategies has been lacking. This review aims to fill this critical gap. The unique positioning and contribution of this manuscript lie in its dedicated synthesis of the flow-physics aspects of laser-produced-plasma extreme ultraviolet (LPP-EUV) sources. Our scope systematically covers: (i) the modeling and simulation efforts related to laser-droplet interactions, covering key aspects such as laser absorption, radiation transport, heat conduction, and material properties; (ii) recent progress in studying the dynamic response of liquid metal droplets under laser pulses, including droplet deformation and target shaping strategies; and (iii) the flow physics of debris migration, discussing debris formation mechanisms, mitigation strategies such as dynamic gas locks, and numerical and experimental methods for analyzing debris transport in low-pressure environments. We expect that this review will interest both the laser-plasma and multiphase fluid dynamics communities, and also stimulate the development of novel strategies for high-efficiency, debris-mitigated EUV light sources.

## OVERVIEW OF PHYSICAL PROCESSES AND SCALES

**Figure 1.**
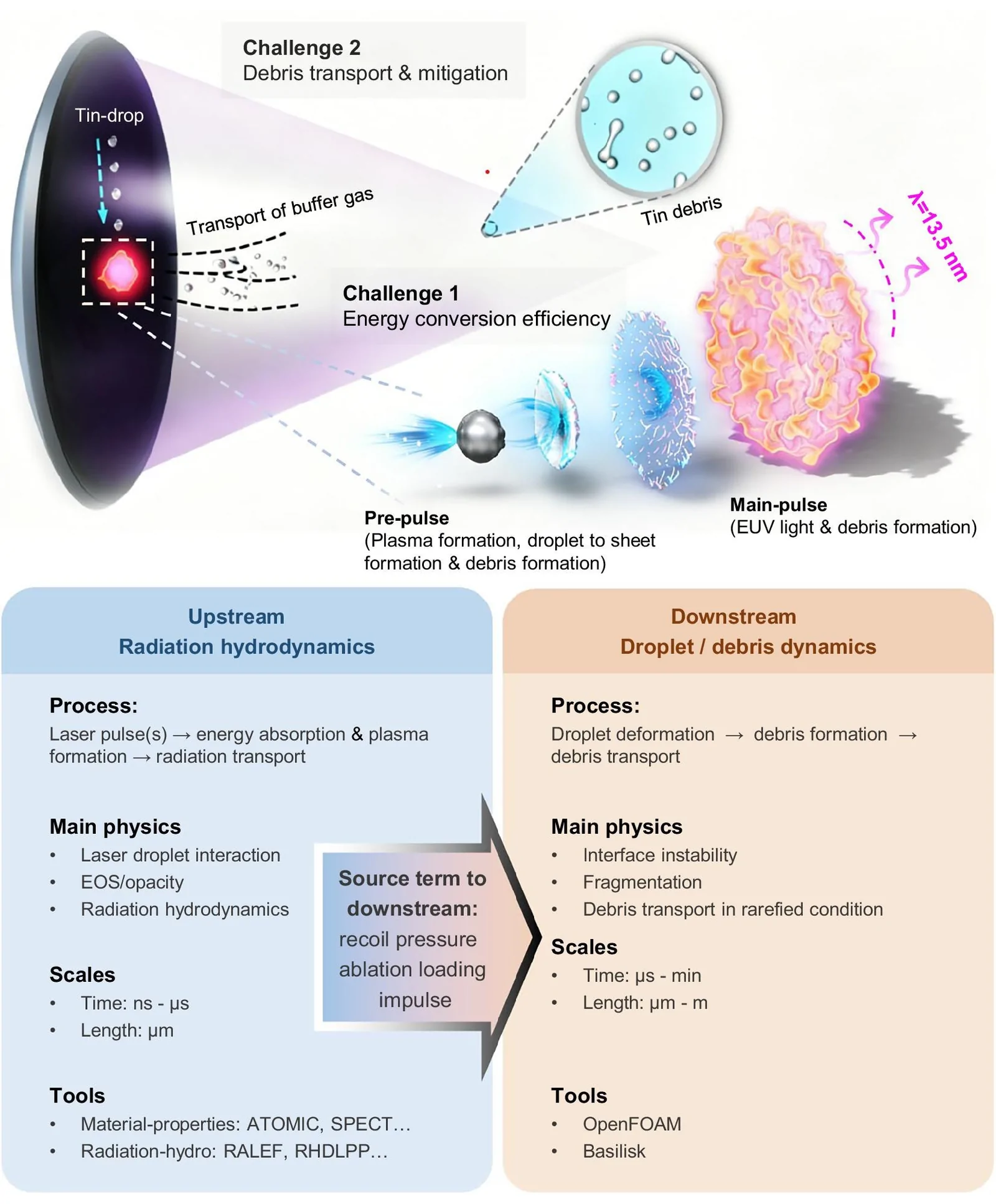
Schematic illustration of the sequential multiphysics processes in a droplet-based laser-produced-plasma extreme ultraviolet (LPP-EUV) source. A falling Sn droplet is first irradiated by a prepulse, which induces ablation, plasma formation, and recoil-driven target shaping. After a controlled delay, a main pulse interacts with the preformed target, leading to intense plasma formation, in-band EUV emission, and debris generation. The upstream stage is dominated primarily by radiation hydrodynamics and material-property modeling, whereas the downstream stage is governed mainly by droplet/debris dynamics and particle-laden transport in rarefied gas. This figure is partially adapted from Ref. [3].

Droplet-based laser-produced-plasma (LPP) EUV sources are inherently multiscale and multiphysics systems, in which laser absorption, plasma formation, droplet deformation, radiation, and debris transport are tightly coupled, as illustrated in Fig. 1. In a typical source, a micrometer-sized molten Sn droplet is first reshaped by a low-energy prepulse, which induces localized ablation and recoil-driven hydrodynamic response and thereby transforms the initially spherical droplet into an expanded preformed target. After a controlled delay, a high-energy main pulse interacts with this target and generates a hot dense tin plasma that emits in-band EUV radiation within 13.5 nm $\pm \, 1\%$. In parallel, both target expansion and plasma evolution produce debris, including ions, neutral clusters, and liquid fragments, which must be mitigated to preserve collector lifetime and source stability.

In the prepulse stage, the dominant physics is the conversion of early plasma loading into droplet-scale hydrodynamic response. A defining feature of this stage is the strong separation of timescales. The relevant hierarchy includes the plasma-hydrodynamic timescale $t_{\rm h}$, the liquid acoustic timescale $t_a$, the laser-pulse duration $t_{\rm L}$, the inertial deformation timescale $t_{\rm i}$, and the capillary timescale $t_{\rm c}$, which satisfy [8–10]


(1) \begin{eqnarray*}
t_{\rm h} < t_{\rm a} < t_{\rm L} \ll t_{\rm i}< t_{\rm c}.
\end{eqnarray*}

Here, $t_{\rm h}$ characterizes the early plasma expansion and pressure buildup, $t_{\rm a}$ is the time for an acoustic disturbance to propagate across the liquid droplet, $t_{\rm L}$ is the laser duration, $t_{\rm i}$ describes the recoil-driven deformation of the droplet, and $t_{\rm c}$ is the timescale on which surface tension becomes dominant. This ordering refers specifically to the prepulse-induced plasma loading and the subsequent droplet hydrodynamic response, rather than to the entire source cycle. For a typical nanosecond prepulse with $t_{\rm L} \sim 10^{-8}$ s [11], plasma forms on an ultrafast timescale $t_{\rm h} \sim R_0/c_{\rm s} \sim 10^{-10}$ s [8], where $R_0 \sim 10,\mu \mathrm{m}$ is the initial droplet radius and $c_{\rm s} \sim 10^5\, \mathrm{m\, s^{-1}}$ is the sound speed in the laser-induced plasma corona. The resulting ablation pressure then drives droplet acceleration and deformation over a much longer inertial timescale, $t_{\rm i} \sim R_0/U \sim 10^{-6}$ s, where *U* is the droplet propulsion velocity [12,13], while capillary effects govern later retraction and breakup on timescales of order $t_{\rm c} \sim 10^{-5}$ s. In current Sn-droplet sources, the droplet diameter is typically $D_0 \sim 25$–$30\,\mu$m, the injection speed is of order $10^2\,\mathrm{m\, s^{-1}}$, and the repetition rate is several $10^4\,\mathrm{Hz}$ [14]. During target shaping, the lateral size of the expanded target can reach a few $10^2\,\mu$m, whereas the stretched liquid sheet may thin to several tens of nanometers [8,15].

In the main pulse stage, the preformed target is converted into a hot dense EUV-emitting plasma. Representative plasma parameters are an electron temperature $T_{\rm e} \sim 20$–40 eV and an electron density $n_{\rm e} \sim 10^{25}\,\mathrm{m^{-3}}$, corresponding to pressure scales of order $n_{\rm e} k_{\rm B} T_{\rm e} \sim 10^7$–$10^8$ Pa, where $k_{\rm B}$ is the Boltzmann constant [8]. The associated Debye length $\lambda _{\rm D}$, which characterizes the electrostatic screening length in the plasma, is of order 10 nm, and the electron plasma frequency $\omega _{\rm pe}$, which characterizes the natural oscillation frequency of the electron component, is of order $10^{14}$ s$^{-1}$ [8]. These values place the main pulse interaction in a hot, dense, transient plasma regime in which laser absorption, radiation transport, ionization kinetics, equation of state (EOS), and opacity are all essential.

The debris stage differs qualitatively from the preceding two stages in both scale and physical regime. Here, the relevant species include ions, atoms, clusters, and neutral droplets or fragments produced by target breakup and plasma expansion. The corresponding transport process extends from the droplet scale to the chamber scale and often takes place in a hydrogen buffer gas of order $10^2$ Pa, for which the gas mean free path is of order $10^{-4}$ m [3]. Under such conditions, chamber-scale flow may remain continuum-like, whereas micron and submicron debris particles experience strongly rarefied conditions. This coexistence of continuum-scale gas flow and particle-scale rarefaction further distinguishes the debris stage from the prepulse and main pulse stages.

Taken together, these large differences in timescale, thermodynamic state, and degree of rarefaction make droplet-based LPP-EUV sources a particularly challenging multiscale problem, in which physically distinct regimes must be understood and modeled in a coordinated manner. This is precisely what makes the field both fundamentally rich and technologically important.

## MODELING AND SIMULATION OF LASER-DROPLET INTERACTIONS

Accurate simulation of the LPP-EUV relevant laser-droplet interactions requires advanced physical models and numerical methods. Simulating such a scenario where lasers interact with a mid-to-high-charge-state and strongly radiating plasma has many aspects in common with multi-physics simulations for inertial confinement fusion (ICF) [16]. Precise modelings on laser-plasma interaction and the radiative properties of plasmas in non-local-thermodynamic-equilibrium (non-LTE) states are always challenging in both LPP-EUV and ICF regimes. While less intense lasers ($I \le 10^{11}$  $\rm {W{\rm cm}}^{-2}$) used in the LPP-EUV regime appear to produce milder laser-plasma environments than those in ICF with laser intensities $I\sim 10^{14}$  $\rm {W{\rm cm}}^{-2}$, lower temperatures in fact complicate the atomic kinetics in tin plasmas. Moreover, the hydrodynamic evolution of tin existing in the liquid phase for quite a long period after the prepulse is also a crucial process to be included in LPP-EUV modelings, as it is now well accepted in the community that the target preparation using a prepulse is critical to both achieving high CE and reducing the debris contamination [8].

Despite that the time scales ranging over six orders of magnitude bring formidable challenges to an integrated simulation accurately taking care of all of the physical processes, the separation of time scales facilitates analyzing the physics and developing simulations focused on specific processes. Consequently, diverse simulation codes have made great contributions to LPP-EUV simulations in different aspects.

**Figure 2.**
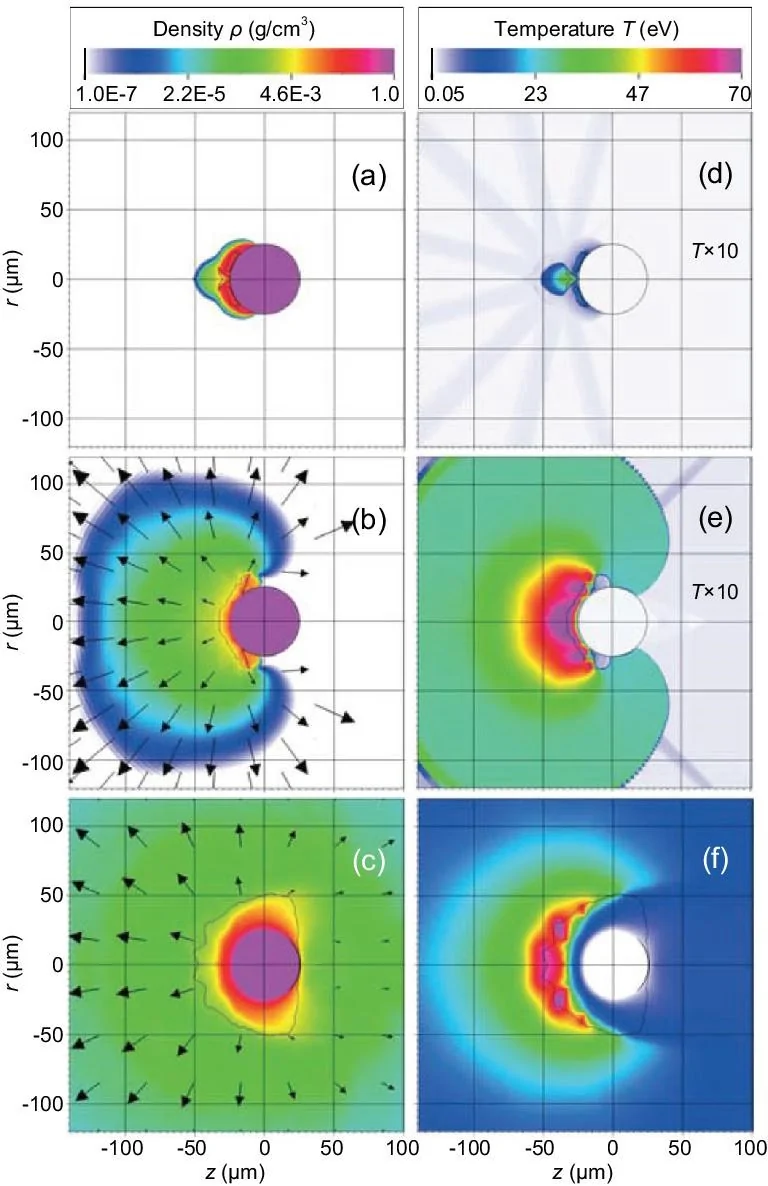
Three simulation results using RALEF-2D code in terms of density and temperature contours: $E_{\rm od}=0.06$ mJ (a and d), 0.2 mJ (b and e), and 30 mJ (c and f), and $d_{\rm foc} =115$  *μ*m at *t = 15* ns when the laser power peaks, where $E_{\rm od}$ is the energy within the original droplet diameter, and $d_{\rm foc}$ is the focus diameter. The black curve is the isocontour of the free electron density $n_{\rm e} = 0.1 n_{\rm cr} = 10^{20}$. Black arrows in (b) and (c) indicate the velocity field in the outflowing plasma. Reproduced from Ref. [33].

One type of codes, such as ATOMIC [17], Cretin [18], Hullac [19], JATOM [20], MPQEOS [21], SEMILLAC [22], SPECT [23], and THERMOS [24], are dedicated to calculating atomic kinetics and radiative properties of tin plasmas in LPP-EUV relevant regimes; these are termed material-property codes. Another type focuses on integrated hydrodynamic simulations incorporating atomic kinetics data and radiation transport; these radiation-hydrodynamic codes include HEIGHTS [25], RZLine [26], RALEF [27], STAR [28], FLASH [29], and RHDLPP [30]. For example, the RALEF code has found extensive application in EUV source modeling in recent times and a set of representative simulation results are illustrated in Fig. 2. The radiation hydrodynamic simulations usually require the material-property codes provide proper EOS and radiation parameters. A third category employs traditional hydrodynamic codes such as OpenFOAM [31] and Basilisk [32], to simulate droplet deformation and fragmentation evolving in the long-term $t_{\rm i}$ or $t_{\rm c}$ scales.

Differences in how the codes model key physical processes, such as the radiative properties of the plasma, EOS, and the absorption and refraction of laser light, lead to deviations in their results [34]. Workshops such as the EUV Light Sources Code Comparison [35], and Non-LTE Code Comparison [36] have advanced code benchmarking and physics understanding. The methodologies for dealing with each key physical process are briefly summarized below.

### Laser absorption

Laser-material interactions in LPP-EUV include multiple stages that involve distinct physical processes. The prepulse first shines on the surface of the liquid tin and undergoes very high reflection. The transient high reflection is physically similar to the “glint” occurring when lasers start shining on the gold surface of an ICF hohlraum [37], thus this early phase when the laser is able to directly reach and reflect on the liquid tin surface is referred to as the glint regime hereafter. As the laser energy is deposited, material vaporization and ionization progressively generate plasma. The critical density $n_{\rm cr}$ formulated as


(2) \begin{eqnarray*}
n_{\rm cr} = \frac{4\pi e^2}{\omega _0^2 m_{\rm e}} \simeq \frac{1.1 \times 10^{21} }{\lambda _{{\mu } \mathrm{m}}^2} \mathrm{cm}^{-3},
\end{eqnarray*}

which is a key concept for laser plasma interactions, where $\omega _0$ is the laser frequency, $\lambda _{\mu \mathrm{m}}$ is the laser wavelength in vacuum in the unit of microns, $m_{\rm e}$ is the electron mass, and *e* is the charge of an electron. A laser with $\omega _0$ is not able to propagate in a plasma with the electron number density above $n_{\rm cr}$. As a plasma denser than $n_{\rm cr}$ forms in front of the droplet, the laser is unable to directly shine on the droplet surface any more but has to deposit its energy into the plasma. Consequently, the remainder of the laser pulses interacts with the generated plasma, leading to the ablation regime [9] which essentially covers the majority of the prepulse and the whole main pulse. An ablation front formed in the plasma is a key feature of the ablation regime. Equation (2) also demonstrates that a shorter-wavelength laser is able to reach higher density inside a plasma. The deeper penetration and also higher absorption are key reasons why solid-state lasers with the wavelengths of 1–2 *μ*m are being considered for next-generation drivers [8,38,39].

Modelings on laser absorption are typically required to supply energy sources in hydrodynamic simulations. Proper modelings need to describe not only different absorption rates in the different regimes, but also the laser propagation in the fluids.

#### Glint regime

Precise modeling on the laser reflection/absorption from liquid tin demands data for optical constants, usually the refraction index and the extinction coefficient, within the LPP-EUV relevant temperature range. The very high reflection from liquid tin is considered to be the key reason for the low CE in the CO$_2$-laser experiments without prepulse [40]. An important role the low-intensity prepulse plays is to create the plasma at low energy losses due to glint [40]. The optical data of liquid tin, which have been obtained through theoretical calculations using a quantum approach [41] and consistent with the experiments, have been implemented in modern LPP-EUV simulation tools [34]. Following the generation of tin vapor around the droplet, the prepulse interacts with the vapor for a short time before significantly ionizing the vapor into a plasma. The laser absorption in vapor can be well modeled based on the main feature of collision-induced absorption that has a quadratic dependence on density [34]. Despite that the glint regime is a transient phase that occurs at very low laser intensity and is energetically subdominant compared to the ablation regime right after the laser intensity rises, the physics close to or below the ablation threshold is attracting recent research interest [33,42,43]. Investigations into the glint regime not only help advance the understanding of laser-droplet interaction, but may also benefit advanced target preparation utilizing laser vaporization below the ablation threshold [43].

#### Ablation regime

Laser absorption in a significantly ionized plasma is dominated by inverse Bremsstrahlung (IB), through which the laser photons are absorbed by the electrons in a plasma while the electrons then collide with the ions. Consequently, the laser energy is transferred into the internal energy of the plasma, thereby heating the plasma [44]. Generally speaking, IB is the dominating laser absorption mechanism from the energetic point of view as the interactions with liquid or vapor of tin are expected to be important only within a short period of the prepulse when the laser intensity is very low. That is likely a reason why laser-liquid or laser-vapor absorption was unconsidered in many works [45]. A commonly used IB laser-absorption coefficient *α* is formulated [35,46] as


(3) \begin{eqnarray*}
\alpha &\equiv& -\frac{1}{I} \frac{\partial I}{\partial z} \\
&=& 900 \left(\frac{n_{\rm e}}{10^{20}}\right) \left(\frac{n_{\rm i}}{10^{20}}\right)\frac{(Z^{*})^2 \lambda _{\mu {\rm m}}^2}{T_{\rm {eV}}^{3/2}}\\
&&\times \, \frac{\ln \Lambda }{\sqrt{1-\frac{n_{\rm e}}{n_{\rm cr}}}} (\mathrm{cm}^{-1}),
\end{eqnarray*}

where *α* describes the fraction of the absorbed laser intensity per unit distance along its path, which is convenient to be implemented in a ray-tracing model that is discussed shortly. $n_{\rm e}$ and $n_{\rm i}$ are the number densities of the electrons and the ions, respectively, $T_{\rm {eV}}$ is the electron temperature in the unit of eV, $Z^{*}$ is the effective charge state (*Z*) of the plasma. Differences in laser absorption among codes usually come from the specific formulations on the Coulomb logarithm $\ln \Lambda$ [35]. Moreover, an anomalous IB absorption reduction due to non-Maxwellian distributions of the electrons on the laser paths, well-known as the Langdon effect [47] in ICF, has also attracted interest in the LPP-EUV regime. A 10%–20% absorption reduction due to the Langdon effect was theoretically predicted [48]. Experimental confirmation of the presence of non-Maxwellian distributions in EUV-source plasmas is therefore desirable.

If the laser pulse duration $t_{\rm L}$ is larger than the hydrodynamic time scale $t_{\rm h}$ of the ablation flow [9] and if the target has adequate mass to sustain the ablation [49], a quasi-stationary ablation front is formed, where the structure of the ablation front only slowly varies in time. A quasi-steady ablation front is a typical feature with nanosecond laser pulses, as demonstrated by simulations [45,50,51] and theory [9]. Simulations also demonstrated that low-mass targets cannot supply enough mass over the entire pulse, therefore leading to reductions in in-band emission (that is, within 13.5 nm $\pm \,1\%$) power and CE [49].

#### Laser propagation

Ray tracing based on geometric optics is a widely adopted method for modeling laser propagation in plasma [52]. This model represents the incident laser with numerous elementary rays, each carrying a fraction of the laser energy and depositing it gradually along its path into the ambient fluid. The attenuation of each ray is governed by the applicable laser absorption model, while its trajectory is a characteristic of the eikonal (Hamilton–Jacobi) formulation. Across computational cell interfaces, the trajectory remains continuous but undergoes a physically-consistent bending governed by the Snell’s law. First-order refraction occurs solely at cell interfaces, while second-order methods incorporate refraction within cells [52]. Many codes [29,30] utilize second-order algorithms derived from Kaiser’s “cell-AVG” method [52], which expresses ray trajectories analytically as quadratic functions, allowing efficient calculation of cell entry and exit points. A limitation of cell-AVG is the iterative procedure required to find entry/exit points in cylindrical or spherical grids [29]. Consequently, a rasterization-based ray-tracing method has been developed [53] to facilitate curvilinear grids by using a geometry-independent numerical formulation for ray trajectories and has removed the requirement to find points on the cell interfaces.

However, the applicability of geometric-optics-based ray tracing is violated in the vicinity of the critical surface. Ray tracing is not able to describe diffraction or “field tunneling” beyond its turning-point density, which directly leads to resonant absorption [54], an anomalous absorption mechanism other than IB. Ray tracing also typically neglects laser polarization. Consequently, wave optics approaches are employed, modeling laser propagation via the Helmholtz equation. Although this time-independent wave equation avoids resolving the laser period, it does require resolving the laser wavelength. This resolution requirement is usually prohibitively expensive for full-scale, high-dimensional simulations. Hybrid approaches have been developed to take advantage of both approaches: geometric optics is used in plasma regions with electron densities below the critical electron density, while wave optics is used close to the critical surface [50]. It was demonstrated that the hybrid approach can fairly accurately reproduce the absorption fraction in both the s- and p-polarizations on arbitrarily steep density gradients [50], and can be efficiently employed in two-dimension (2D) and three-dimension (3D) simulations.

### Heat conduction

Heat conduction of plasmas is mostly modeled by the Fourier law that the heat flux $\vec{q}_T$ is proportional to the negative gradient of the temperature, that is,


(4) \begin{eqnarray*}
\vec{q}_T = -\kappa _T \nabla T,
\end{eqnarray*}

where the heat conduction coefficient $\kappa _T$ is highly nonlinear to the plasma temperature *T*. $\vec{q}_T$ is then incorporated in the energy equation of the radiation hydrodynamic equations. The classical Spitzer–Harm (SH) model [55] shows that $\kappa _T \propto T ^{5/2}$ in a plasma, and thus $\kappa _T$ varies by orders of magnitude between hot plasma and liquid tin within the hydrodynamic system. In radiation hydrodynamic simulations, $\kappa _T$ is usually taken from tabulated data that are generated by a material-property code, for example, THERMOS [24].

The Fourier law in the form of Eq. (4) is often referred to as the local heat conduction, that is, the heat flux only depends on the local hydrodynamic status. However, the temperature gradients can sometimes be sufficiently large that the assumption of the SH model that the mean free path of high-energy heat-carrying electrons is much smaller than the scale length of the temperature gradient breaks down in such regimes. In these cases, the non-local heat transport should be considered, which has been well-recognized in ICF utilizing significantly intenser lasers than LPP-EUV [56]. An *ad hoc* heat flux limiter [57] is often employed to take account of flux mitigation but it is unable to capture preheating physics. The nonlocal multigroup diffusion model known as the SNB model [58], which balances physical accuracy and computational efficiency, has been widely used for non-local heat conduction in ICF-relevant hydrodynamic simulations [56]. Effects due to non-local heat transport might become significant in next-generation LPP-EUV schemes using intenser lasers.

### Radiation transport

Radiation transport in complex plasma environment is crucial for understanding plasma evolution and EUV generation. The modeling of radiation for LPP-EUV involves two levels of requirements from different points of view: (i) spectroscopically, high-resolution simulated spectra capturing detailed line structures near 13.5 nm are essential for experimental validation, though this region is dominated by numerous unresolved transition arrays [59]. Achieving such accuracy relies on sophisticated atomic physics and remains inaccessible even for advanced first-principles atomic codes [8] (progress in semi-empirical methods is beyond this scope and can be found in Ref. [8]). (ii) Hydrodynamically, coarser modelings but with the capability of describing the key radiation effects, for example, radiation cooling and energy coupling with hydrodynamics, are usually adequate. Here, radiation transport plays a similar role as the heat transport, but in a nonlocal manner due to large mean-free-paths of photons. Radiation transport can also be one of the main mechanisms responsible for the target heating and vaporization [9,34]. Then spectra with finer resolutions can be produced by post-processing the hydrodynamic simulation data [60] using a spectroscopic code, for example, FLYCHK [61], CRETIN [18], and SPECT [23].

Modeling radiation transport in hydrodynamic codes requires balancing accuracy with computational efficiency when solving the radiation transport (RT) equation [62]:


(5) \begin{eqnarray*}
\frac{1}{c} \frac{\partial I_\nu }{\partial t} + \vec{n}\cdot \nabla I_\nu = \eta _\nu - \kappa _\nu I_\nu ,
\end{eqnarray*}

where $I_\nu (\vec{r},\vec{n},t)$ is the high-dimensional spectral intensity, that is, the energy crossing a unit area at a given point $\vec{r}$ with a certain frequency *ν* per unit time per unit frequency and per unit solid angle in the direction denoted by the unit vector $\vec{n}$. The coefficients $\eta _\nu$ and $\kappa _\nu$ denote the spectral emissivity and opacity that depend on the plasma or material properties (mostly the electron temperature and density), respectively. Note that Eq. (5) neglects scattering effects.

#### Diffusion models

The simplest approximation to Eq. (5) is a diffusion model that is valid for optically thick regimes, that is, $\kappa L \gg 1$, where *L* is a characteristic length and *κ* is a typical opacity of the system. Instead of solving for $I_\nu$, diffusion models solve for its angular moments. A diffusion model assuming that the radiation photons obey the blackbody spectral Planckian distribution is usually referred to as the gray diffusion model [62]. Integration over the frequency and the solid angle *Ω* converts Eq. (5) into a diffusion equation that solves for the radiation energy density $E_{\rm r}$ defined as


(6) \begin{eqnarray*}
E_{\rm r} \equiv \frac{1}{c} \int _{0}^{\infty } \mathrm{d}\nu \int _{4\pi } I_\nu \mathrm{d}\Omega .
\end{eqnarray*}

The gray diffusion model is convenient to be implemented in hydrodynamic codes [63] and has also been widely used in LPP-EUV simulations [30,60]. An enhancement is the multi-group diffusion model, which discretizes the frequency domain into energy groups (*g = 1, 2, 3,*...), each characterized by its own radiation energy density $E_{\rm g}$. This approach allows moderate deviations from the Planckian distribution and is able to provide spectral resolution [64].

For optically thin and intermediate regimes ($\kappa L \le 1$), similar to the flux limiter for heat conduction, flux-limited diffusion approaches [64–66] can provide phenomenological modeling by modifying diffusion coefficients to restrict the effective photon speed (lower than the speed of light). However, results obtained by diffusion models in these regimes, which do exist in the low-density corona plasmas of LPP-EUV, might be questionable. Transport models solving the RT equation Eq. (5) for the spectral intensities rather than the energy-density formulations ($E_{\rm r}$ or $E_{\rm g}$) provide more self-consistent descriptions on radiation in these regimes.

#### Transport models

One type of widely-used deterministic methods solve the RT equation along discrete angles and are often referred to as the discrete ordinates ($S_\mathrm{N}$) methods [62]. A popular treatment known as the variable Eddington tensor (VET) scheme uses the quasi-static formal solution to the RT equation as a closure to the radiation moment equation to reduce the numerical cost, by decoupling the time-dependent evolution of radiation hydrodynamics and the time-independent radiation transport [67]. However, efficiently solving the full time-dependent RT equation in a finite volume approach was also demonstrated to be practically achievable [68]. A disadvantage of $S_\mathrm{N}$ methods is known as the “ray effect” that sometimes produces non-physical streaks in solutions [62]. Another type of method is based on a Monte Carlo (MC) solution methodology. In particular, a popular method known as the implicit MC [69] has been widely used in many fields, including astrophysics and ICF. Novel schemes have also been developed to reduce the noise and computational cost of MC methods in different regimes in recent years [70,71]. Both $S_\mathrm{N}$ [49] and MC methods [34] are employed in LPP-EUV simulations. Special weight factors have been developed to enhance the accuracy and speed of the calculations for MC in the special LPP-EUV regime [34].

### Material properties: equations of state and radiation properties

The fidelity of all radiation modeling approaches discussed above relies on the quality of atomic physics data such as tabulated opacity and emission coefficients. Uncertainties in material properties, including these radiation coefficients and the equations of state (EOS) for tin plasmas and droplets, complicate simulations as well. Tin exists in various states of plasmas and liquid, which have distinct densities and temperatures. These complex behaviors are rather difficult to capture with a universal EOS. Moreover, in contrast to the LTE regime where the EOS of material is independent of the radiation environment, most LPP-EUV plasmas under conditions of interest in practical applications are in the non-LTE regime [72], that is, in principle the plasma properties should be written as functions of temperature, mass density, and local spectral radiation energy density, and time.

To address these challenges, the Frankfurt EOS (FEOS) [73] has been widely used in LPP-EUV works [30,33,39,45,49,60], and provides hydrodynamic codes with reliable EOS data for a broad range in temperature-density space. Building upon the well-established QEOS model [74] and evolving from the MPQeos code [21], FEOS incorporates a remarkable feature: explicit treatment of the liquid–gas phase transition in the low-temperature regime using Maxwell’s construction for fully equilibrated two-phase regions. This approach has proven effective for modeling phenomena like explosive boiling induced by ultra-short laser pulses [75].

Knowledge of atomic-level population distributions of particles is also the key to quantifying radiation properties including opacity and emissivity, which are closely related to thermodynamic parameters such as temperature and density. Different plasma models, for example, thermal equilibrium (TE), LTE, coronal, and collisional radiative (CR), are applicable to different parameter regions [61]. For LPP-EUV hydrodynamic simulations, the CR model is often used in the material-property codes such as THERMOS [24] and SNOP [76] to prepare the opacity data in tabulated form beforehand in recent works [39,60]. It is important to note, however, that obtaining reliable opacity data for high-Z elements like tin is non-trivial due to their inherent sensitivity.

## HYDRODYNAMIC RESPONSE OF LASER-DRIVEN TIN DROPLETS

The previous section focused on the modeling framework for early-time laser energy deposition, including thermophysical properties, radiation transport, and plasma evolution. We now turn to the subsequent droplet-scale response, which bridges early-time laser-plasma interaction and the later main pulse coupling in droplet-based LPP-EUV sources. At this stage, the central issue is no longer the detailed plasma kinetics themselves, but rather how prepulse- and short-pulse irradiation reshape the initially spherical tin droplet into a favorable target for the main pulse [77]. This hydrodynamic response involves target shaping, cavitation, shell or sheet expansion, fragmentation, and the onset of debris generation, and therefore plays a pivotal role in determining both conversion efficiency and downstream debris behavior [11].

### Prepulse-controlled target shaping

In droplet-based LPP-EUV sources, the prepulse affects CE primarily through target preparation rather than through direct generation of the in-band EUV-emitting plasma. Its function is to reshape the initially spherical tin droplet into a preformed target with an appropriate lateral extent, thickness distribution, aspect ratio, and axial position at the instant of main pulse arrival. Because the coupling of the main pulse depends sensitively on this target state, prepulse optimization has become a central route toward improving CE. At the same time, the same target-shaping strategies also influence debris generation, so that CE enhancement and debris mitigation must be considered in a coupled manner.

**Figure 3.**
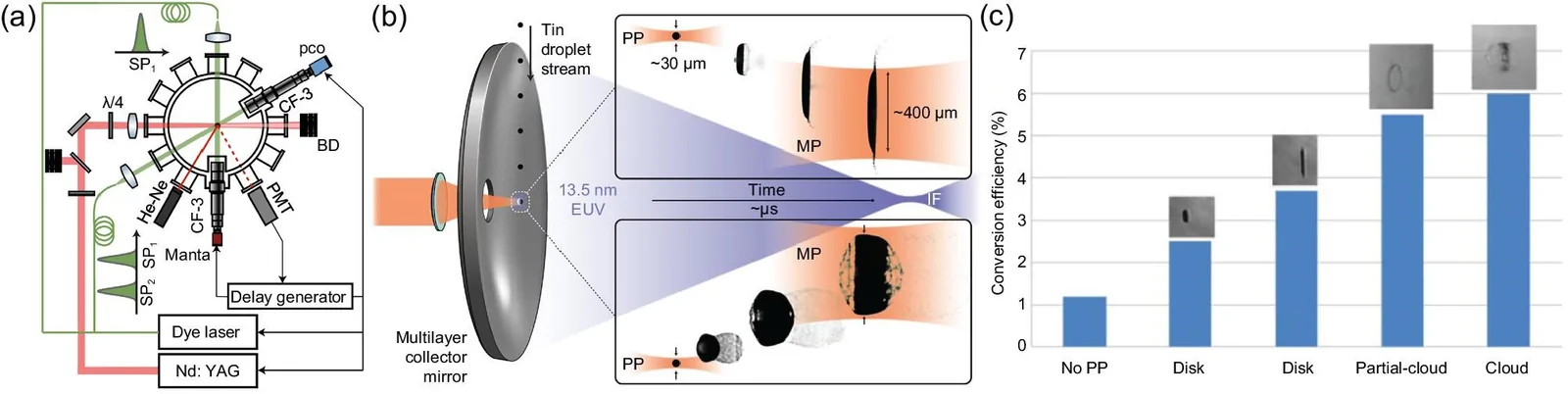
Prepulse-controlled target shaping and its implications for conversion efficiency. (a) Top-view schematic of the experimental setup for laser-pulse impact on a liquid metal droplet. (b) Illustration of the laser-droplet-based EUV source operation principle with a prepulse (PP) followed by a main pulse (MP). (c) The relation between the conversion efficiency and the target state after prepulse impact. Panel (a) reproduced from Ref. [78]; (b) reproduced from Ref. [77]; and (c) reproduced from Ref. [11].

A representative experimental configuration is shown in Fig. 3a [78]. For nanosecond prepulses, laser ablation on the irradiated side produces a transient tin plasma, which exerts a recoil-pressure impulse on the remaining liquid and drives the droplet into an expanding disk-like target [10,13]. After a delay of a few microseconds, the main pulse is then applied to this preformed target, as illustrated schematically in Fig. 3b [77,79]. In this regime, prepulse design is essentially a problem of controlled target shaping.

When picosecond or femtosecond pulses are employed, the droplets undergo bubble-like expansion, forming target structures referred to as “dome” or “cloud” targets [80], as shown in the bottom row of Fig. 3b. Such volumetrically shaped targets, depicted in Fig. 3c, have demonstrated significant potential for achieving high conversion efficiency [11], likely owing to their extended interaction volume. However, they also introduce additional challenges in debris mitigation, since spallation can generate fast neutral debris [81,82].

Beyond the conventional single-prepulse configuration, advanced target-shaping strategies have become an active research direction in both fundamental studies and source optimization [77,83]. These approaches include partial vaporization or rarefaction of the preformed tin target, preheated-plasma formation followed by laser reheating [15,84,85], as well as double- and multi-pulse target preparation and temporal shaping of the prepulse itself [86,87]. Their shared objective is to tailor the target morphology, mass redistribution, and spatial configuration prior to main pulse irradiation, thereby improving laser–target coupling and potentially increasing the in-band CE [77,86]. At the same time, these strategies often exacerbate cavitation, spallation, and neutral-debris production, highlighting the need for a coupled optimization of target shaping and debris control [15,87].

### Experimental advances in droplet dynamics

**Figure 4.**
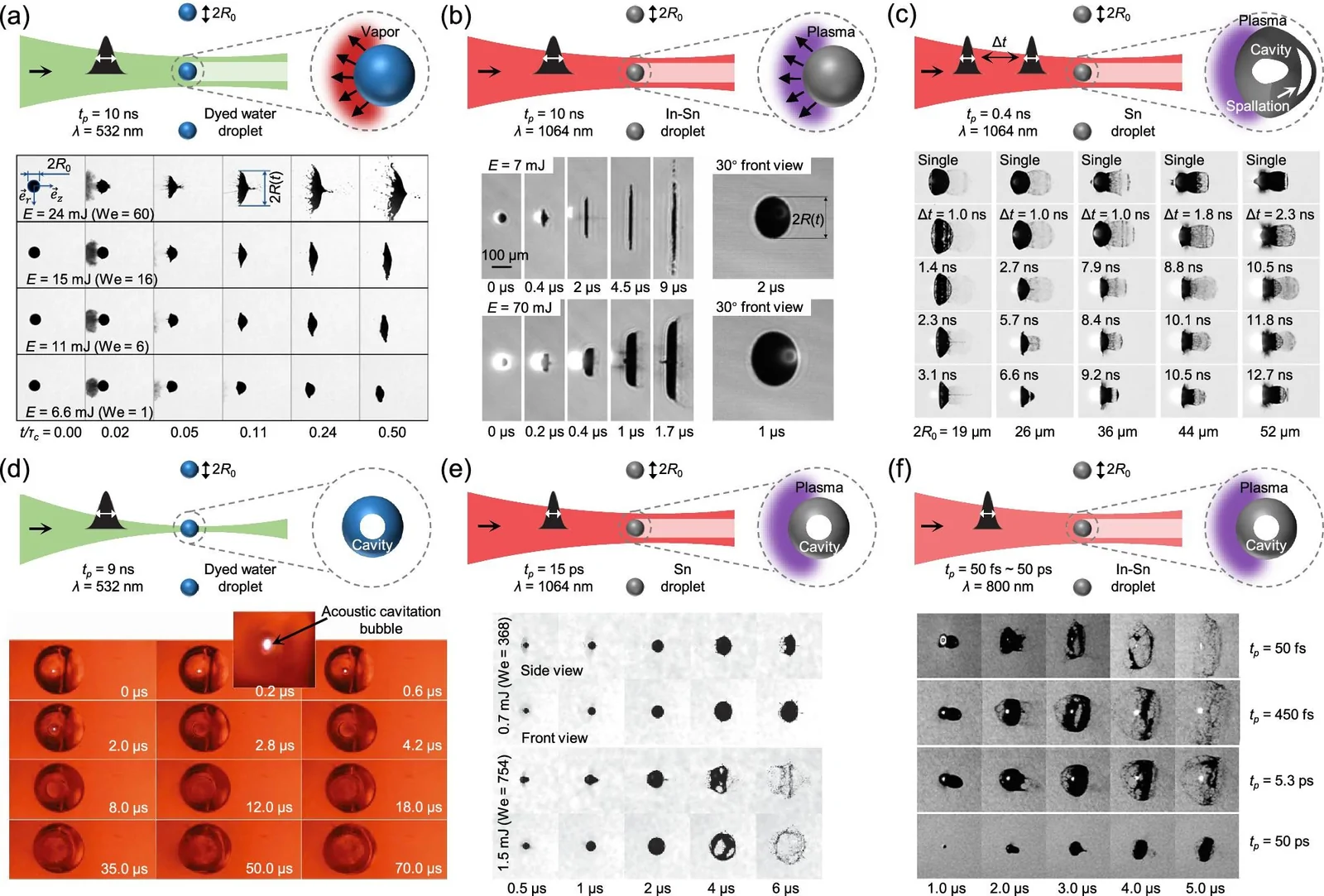
Droplet dynamics under prepulse impact. (a) Nanosecond-laser-pulse impact on a dyed water drop. (b) Nanosecond-laser-pulse impact on an In-Sn droplet. (c) Double-nanosecond-laser-pulse impact on a Sn droplet. (d) Focused-nanosecond-laser-pulse impact on a dyed water drop. (e) Picosecond-laser-pulse impact on a Sn droplet. (f) Femtosecond-laser-pulse impact on an In-Sn droplet. In panels (c, e and f), the plasma is schematically shown only on the laser-irradiated side of the droplet; the rear-side response arises from pressure-wave propagation, cavitation, and/or spallation rather than direct plasma formation. Panel (a) adapted from Ref. [12]; (b) adapted from Ref. [13]; (c) adapted from Ref. [91]; (d) adapted from Ref. [92]; (e) adapted from Ref. [82]; and (f) adapted from Ref. [93].

The hydrodynamic response of laser-driven droplets has been investigated extensively over the past decade, motivated both by its fundamental fluid-mechanical richness and by its practical importance for target shaping in LPP-EUV sources. Experimental studies have focused in particular on the roles of pulse duration, laser wavelength, pulse energy, focal geometry, and droplet material. Early studies examined the impact of laser pulses on dyed water droplets [12], providing valuable insights for subsequent research on liquid metal droplets [88–90], as illustrated in Fig. 4a. The typical response involves four distinct timescales: (i) energy absorption leading to plasma formation and localized boiling; (ii) material ejection, in which part of the liquid is projected outward at speeds exceeding kilometers per second, fragmenting into numerous tiny droplets, potentially accompanied by shock wave emission; (iii) translation of the droplet’s center of mass due to ejection recoil; and (iv) global-scale droplet deformation.

Some time after irradiation (typically tens of microseconds for nonmetal droplets), the remaining portion of the droplet may deform and spread into a liquid film or further disintegrate into smaller droplets, as shown in Fig. 4a. By analyzing momentum conservation between the ejected vapor and the propelled droplet, a linear relationship was established between the propulsion velocity *U* and the deposited laser energy *E*. The subsequent deformation can then be characterized by the Weber number (We), $\mathrm{We}=\rho R_0 U^2/\gamma$, which compares the kinetic energy associated with droplet deformation with the surface energy. This leads to the scaling law:


(7) \begin{eqnarray*}
\frac{R_{\max }-R_0}{R_0} \sim \mathrm{We}^{1/2},
\end{eqnarray*}

where *ρ* and *γ* denote the density and surface tension of the droplet, respectively, and $R_{\rm max},\, R_0$ are the maximum droplet radius and initial droplet radius, respectively.

In contrast, under nanosecond laser impact, liquid tin droplets exhibit a more characteristic sequence of laser-driven deformation dynamics, as shown in Fig. 4b [13]. First, laser energy is absorbed near the irradiated surface, leading to rapid ionization and plasma formation. The expansion of this plasma generates a recoil pressure (or pressure impulse) on the remaining liquid, which accelerates the droplet and drives it to deform into a radially expanding thin sheet. As the sheet expands, inertia initially dominates, whereas surface tension becomes increasingly important at later times, slowing down and eventually reversing the expansion. During this capillary-controlled stage, the liquid sheet develops azimuthal corrugations and rim fingering, which can further lead to ligament formation, fragment ejection, and breakup into secondary droplets. This sequence, plasma formation, recoil-driven sheet expansion, capillary retraction, and instability-mediated fragmentation, constitutes the basic physical picture of nanosecond laser impact on tin droplets.

Based on a laser-induced mass ablation model, momentum conservation, and extensive experimental data, Kurilovich *et al.* [33] proposed a semiempirical model for the propulsion velocity $U = (E_\mathrm{d}/\tau _\mathrm{p})^{0.6}$, where $E_\mathrm{d}$ is the deposited laser energy and $\tau _\mathrm{p}$ is the pulse duration, and for the expansion velocity $\dot{R}_0 = E_\mathrm{d}^{0.7}$.

Moreover, using a double-prepulse approach, a cavitation bubble can form due to the propagation and interaction of pressure waves inside the droplet [91]. The formation and growth of this bubble leads to the generation of a fine droplet cloud or mist, which may enhance conversion efficiency but complicates debris mitigation, as illustrated in Fig. 4c. More recently, Xu *et al.* [92] employed opaque dye-doped water droplets to visualize internal cavitation and splash dynamics under tightly focused nanosecond laser pulses, revealing mechanisms analogous to those in metallic droplets, as shown in Fig. 4d.

Furthermore, under picosecond laser pulses, the droplet exhibits cavitation-bubble-like expansion, as shown in Fig. 4e [82]. This occurs because the ablation pulse launches a strong compressive pressure wave from the laser-irradiated side of the droplet. Since the pressure loading is applied on a timescale much shorter than the acoustic transit time across the droplet [94], the liquid responds in a stress-confined manner. As the compressive wave propagates inward, it converges toward the droplet center; however, the cavitation observed in the interior is not caused directly by the positive-pressure peak itself. Rather, the unloading/rarefaction wave that follows the compressive pulse is likewise focused toward the center, where it generates a sufficiently large tensile stress to rupture the liquid and form an internal cavity or thin shell. After passing through the center, the wave reaches the rear free surface, where reflection converts the compressive disturbance into an additional tensile pulse, which can induce rear-side rupture and spallation. The subsequent expansion of the cavitation-driven shell can then be described using a Rayleigh–Plesset-type framework [95]. When the laser pulse duration is reduced to femtoseconds, the droplet dynamics remain similar to those under picosecond pulses [93], as shown in Fig. 4f.

### Numerical simulations of droplet dynamics

**Figure 5.**
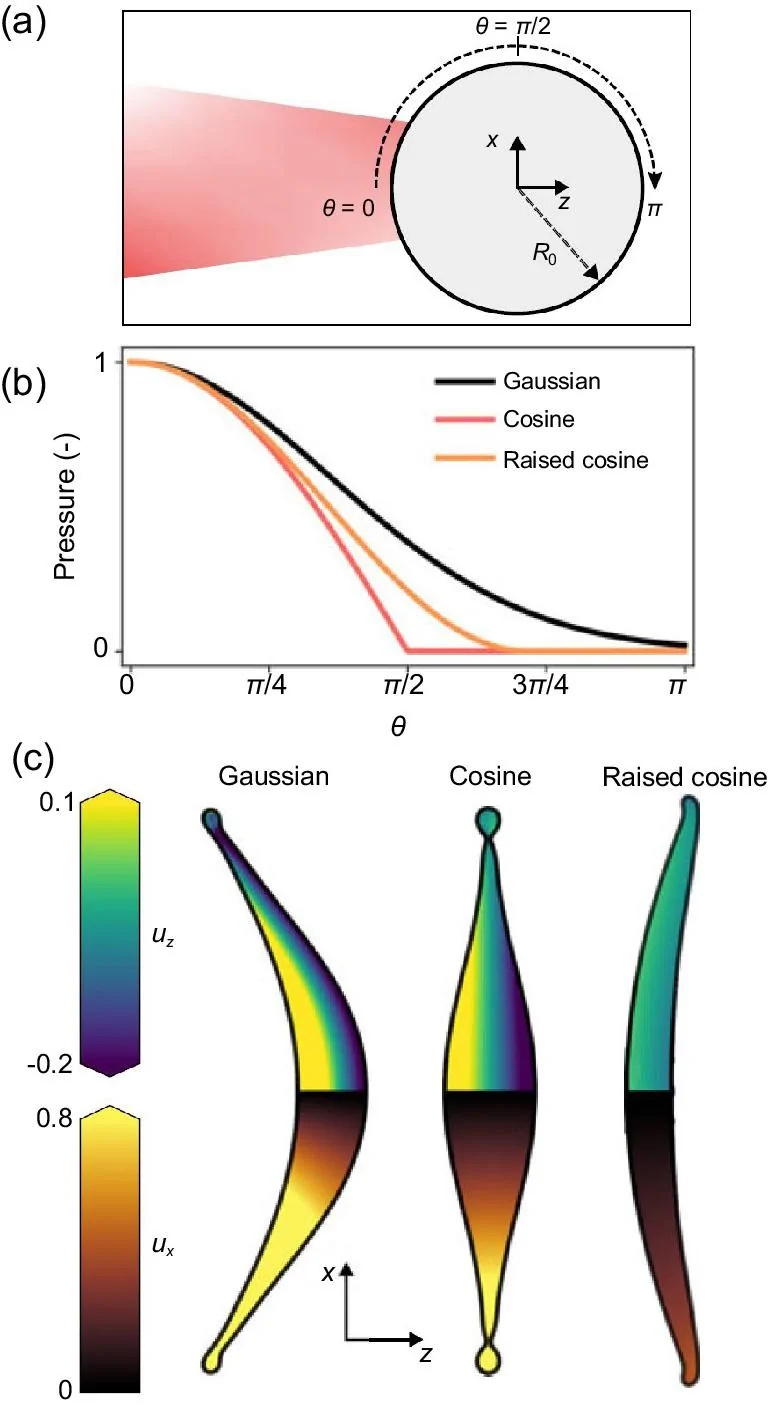
Illustration of deriving a velocity field from a specified surface pressure profile on a droplet. (a) Sketch of the droplet and laser. (b) Examples of possible initial pressure profile functions on the droplet surface. (c) Radial and axial velocity components on three sheets obtained from different pressure profile types. Adapted from Ref. [96].

Numerical simulations focusing only on droplet dynamics can employ a pure hydrodynamic framework decoupled from the laser-plasma interactions and plasma physics. Within this approach, the droplet is usually modeled as incompressible and inviscid, and a simplified ablation pressure mimics the plasma impact [10,12,96]. Deformation of a droplet impacted by a laser pulse was simulated using a boundary integral method coupled with such ablation pressure model [10], and different ablation pressure profiles were found to drive the droplet into distinct shapes. To determine the dependence of droplet deformation on the duration of the pressure pulse at constant total momentum, an analytical acoustic model (assuming small density fluctuations) was further derived for the internal pressure, pressure impulse, and velocity fields [94]. However, a key discrepancy arises: experimentally observed tin sheets in advanced EUV sources often exhibit curvature inversion, which is opposite to initial theoretical predictions [96]. To resolve this, simulations successfully reproduced the inversion using an instantaneous pressure impulse modeled by a low-kurtosis raised cosine function [96], as shown in Fig. 5. Furthermore, fragmentation of an incompressible droplet was investigated via a two-fluid approach with a pressure impulse [97]. The results showed a good agreement with experimental observations of [12] with respect to the expansion of the liquid sheet and the development of a polydisperse cloud of fragments. Recent studies have further elucidated the flow patterns and wave propagation mechanisms inside a droplet subjected to a picosecond laser prepulse. Through simulations of compressible two-phase flows, Li *et al.* [98] analyzed the wave structure of compression-expansion-recompression induced by localized energy deposition and established a theoretical model under the small perturbation assumption, revealing the intrinsic link between droplet deformation and wave propagation.

Although the pressure-impulse modelings offer computational convenience for studying droplet dynamics, they face limitations: the spatial-temporal pressure profiles must be empirically prescribed; moreover, they inherently neglect the dynamic feedback of droplet deformation and motion on the plasma, which is expected to continuously modify the in-flight pressure profile applied to the surface. Therefore, the development of advanced plasma-liquid modeling that can self-consistently handle both the laser-plasma physics and the plasma-liquid interactions is emerging [99] as a critical future direction for simulations of laser-droplet interaction.

## FLOW PHYSICS OF DEBRIS MIGRATION

An inherent challenge associated with the LPP-EUV light source is the unavoidable generation of debris as a byproduct of the laser-plasma interaction. The debris encompasses a variety of species, including highly energetic ions, weakly-charged atoms, and microscopic particles of tin, all ejected from the plasma source [72,100–103].

A thorough understanding of the fundamental principles governing the movement of debris, specifically the flow physics within the EUV vessel, is of paramount importance. Since EUV light of 13.5 nm wavelength can be readily absorbed by air molecules at normal pressure and by all other media, the light source vessel needed to be maintained at low pressure and with the gas with less absorption rate, such as hydrogen [104]. The knowledge on how debris transport in relatively low pressure environment is crucial for the design and optimization of the vessel itself, the implementation of effective mitigation strategies aimed at controlling and removing the debris, and ultimately for ensuring the long-term viability of EUV lithography as a robust technology for high-volume manufacturing of next-generation semiconductor devices.

In this section, we first discuss the mechanisms of debris formation, followed by an analysis of its key characteristics. We then present various debris mitigation strategies. As these strategies are closely linked to the underlying flow physics, we review the numerical approaches used to investigate debris transport under EUV vessel conditions. Finally, we describe the corresponding experimental techniques for measuring flow behavior in such low-pressure environments.

### Debris formation

The deformation of droplet by prepulse and expansion and cooling process of high-temperature tin plasma by main pulse generates a considerable amount of debris [75,81].

The first route of forming debris is from the irradiated plasma: initially, the plasma is quasi-neutral, with equal densities of positive ions (Sn$^{n+}$) and free electrons [105]. The expansion is driven by the pressure gradient from the hot, dense plasma core to the cooler, low-density ambient. The expansion velocity is supersonic with the lighter electrons move faster than the ions. As the plasma expands and cools, electron–ion recombination takes place and thus reduces the charge state of Sn ions [106]. After the cooling down of plasma, there are several types of debris forms: first, the charged debris as the consequence of the partially recombined ions. Second, Sn atoms combine together and condensation takes place which forms the particular debris. Note that the first type of debris involving high energetic ions is destructive. When these fast ions impact the EUV collector, they lead to severe atomic sputtering, surface etching, etc. It irreversibly degrades the surface roughness and drastically reduces the in-band EUV reflectivity [107–110].

**Figure 6.**
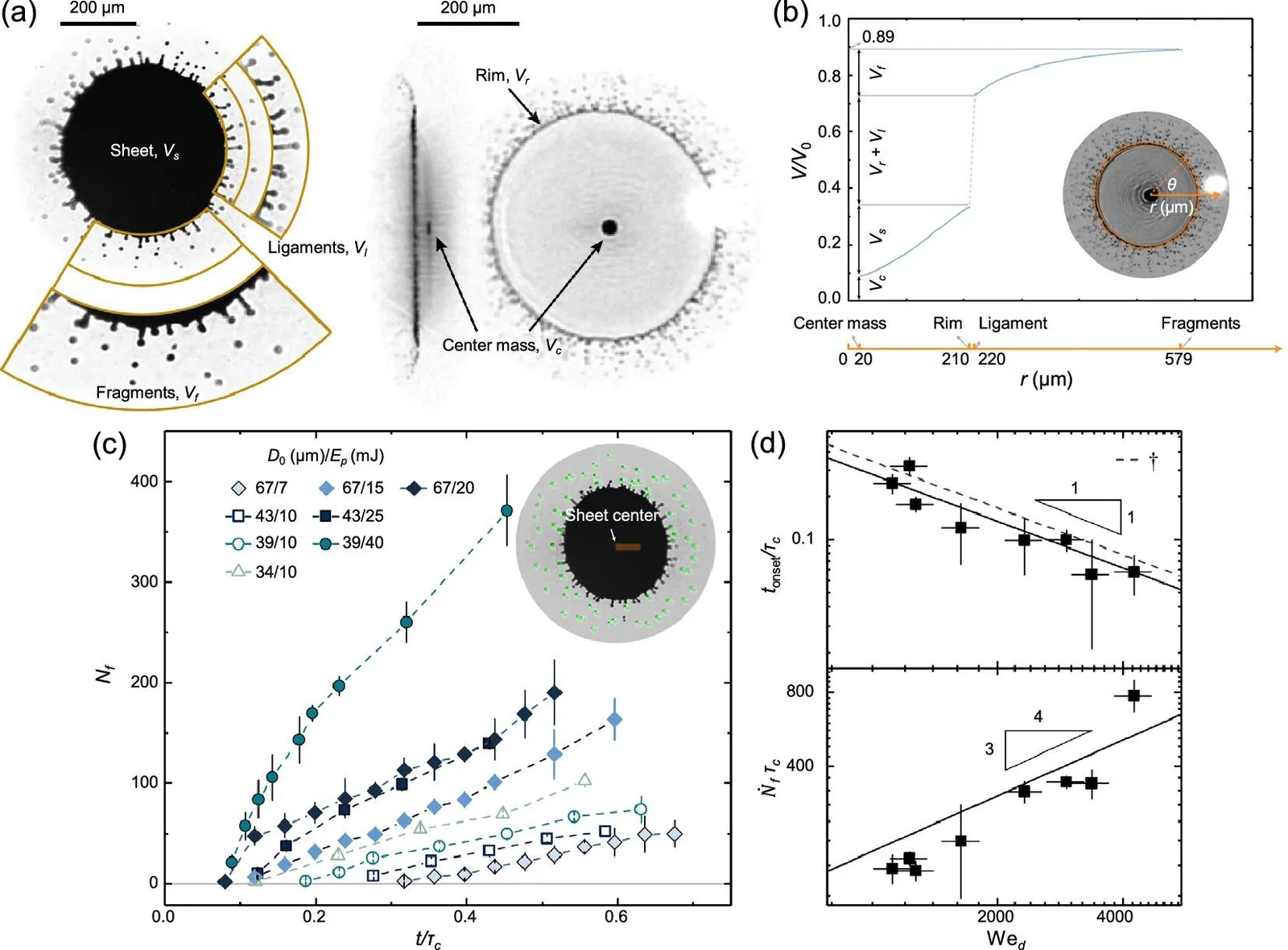
(a) The typical regions of the expanding liquid metal thin film based on their distance from the mass center. (b) The cumulative volume fraction of liquid metal within different typical region. (c) Fragment number at the rim region as a function of non-dimensional time. (d) Onset time of fragmentation and the rate of increase of the fragment number as a function of the Weber number. Adapted from Ref. [78].

The second route of forming debris is from the prepulse process. With the prepulse laser impact on tin droplets, the droplet deforms into sheet-like where there could be substantial liquid away from the laser interaction zone, giving a significant source of neutral debris from sheet fragmentation [5]. As shown in Fig. 6a, the expanding liquid sheet can be divided into five parts based on their distance from the center of the drop: center mass, sheet, rim, ligaments, and fragments [78]. Using optical measurement techniques (for example, transmission signals [111]) and fluid dynamics analysis (for example, Taylor–Culick theory [112]), the researchers have quantified the volume fraction changes in each region, as depicted in Fig. 6b. Furthermore, by studying the number of fragments, their formation time, and the rate of increase (Fig. 6c), the mechanism of ligament fragmentation can be inferred. This mechanism aligns with the theoretical framework of linear instability coupled with adjustment of nonlinear rim thickness in unstable ligament fragmentation [113].

### Debris characteristics

It is important to understand characteristics of the debris in a way to mitigate debris, for example, the composition, density, velocity distribution of these tin debris, and also importantly the charge state.

The research group from University of Illinois at Urbana-Champaign (UIUC) have constructed a series of setup for debris characterization in EUV lithography environment. The so-called Illinois Calibrated Spherical Sector Electrostatic Energy Analyzer have been constructed to provide absolute measurements of ion debris flux. The analyzer further allows identification of individual ion species through energy-to-charge ratio discrimination [114]. The UIUC group has further integrated multiple components—including a Faraday cup, residual gas analyzer, microchannel plates, dual quartz crystal microbalances, and witness plates—to enable comprehensive quantitative and qualitative analysis of undesired flux [115].

The velocity of debris differs significantly between ions and droplets. In particular, energetic ions can reach velocities on the order of tens of kilometers per second [77,116–119], whereas droplets typically exhibit much lower velocities [93,120]. The velocity distribtion of the droplet debris has been experimentally characterized for tin droplet irradiated using femto- and picosecond prepulse [93]. Such debris emission is found to be anisotropic, with the strongest ejection occurring along the laser beam direction (up to 300 m s^−1^), while significantly lower velocities (around 100 m s^−1^) are observed in the perpendicular and opposite directions. There is recent work from Deng group [120] using varying laser power irradiating on gallium–indium–tin liquid film. They show that velocities of droplet debris range from 329 m s^−1^ to 4211 m s^−1^.

Ion debris from laser-produced mass-limited tin plasmas has been experimentally characterized for EUV lithography applications. The study examines scaling laws for optimizing target mass to reduce contamination of the first EUV collection mirror. Measurements show that the ion energy spectrum aligns well with predictions from the isothermal expansion model [121]. Recent studies on the expansion of laser-produced plasmas have shown that the electrons often follow a non-equilibrium kappa distribution rather than a Maxwell–Boltzmann one, and that ion cooling is slower than predicted by the adiabatic law [122]. These non-equilibrium thermodynamic behaviors, which affect ion acceleration and the resultant debris spectrum, must be considered for a comprehensive understanding of the LPP-EUV source lifecycle.

Regarding the charge state of ions, the work by Sheil *et al.* [123] has shown that there exists a scaling relationship between the average charge state and the kinetic energy of the ions, suggesting that complete recombination to neutral atoms is practically unattainable in such the highly transient condition where there persists some low-charge-state ions (such as Sn$^{1+}$, Sn$^{2+}$). Besides, the charge state of ions can be further reduced when tin ions collide with H$_2$ buffer gas. The seminal experimental and theoretical studies by the Hoekstra group [124,125] have provided detailed state-selective electron capture cross sections for highly charged tin ions colliding with molecular hydrogen.

### Flow driven debris mitigation strategies

A direct mitigation strategy is the use of mass-limited droplet targets [126]. In addition, alternative approaches rely on the incorporation of auxiliary components, such as electric field [127], magnetic deflection [104], foil trap [128], hydrogen buffer gas, chemical reactions [129], or the hybrid methods such as combining magnetic deflection with buffer gas [130]. Table 1 provides the overview of major mitigation strategies. Here, we primarily focus on hydrogen buffer gas approaches, as they are closely related to flow dynamics.

**Table 1.** Comparative analysis of primary debris mitigation strategies in LPP-EUV sources, evaluating their effectiveness, impact on EUV transmission, and operational complexity.

Mitigation strategy	Typical reduction efficiency	Impact on EUV transmission	Operational complexity & limitations
Dynamic gas lock (H$_2$ buffer gas)	Efficient for ions, weakly charged particle and tiny droplets (via collisional)	Absorb weakly for small molecular weight, e.g. H$_2$ gas. Also depending on flow rate of H$_2$ buffer gas	Requires complex gas flow control, large pumping capacity, and management of thermal loads transferred to the gas.
Magnetic fields	Very efficient for fast ions deflected from the collector optical path; Ineffective for weakly-charged particles and droplets	Fields do not absorb or block EUV photons	Requires strong, stable electromagnetic systems
Foil traps (physical shields)	Efficient for particles and tiny droplet	Severe due to direct obstruction of the optical path, except with dedicated design	Risk of thermal damage or sputtering, generating secondary debris; often requires active cooling.

**Figure 7.**
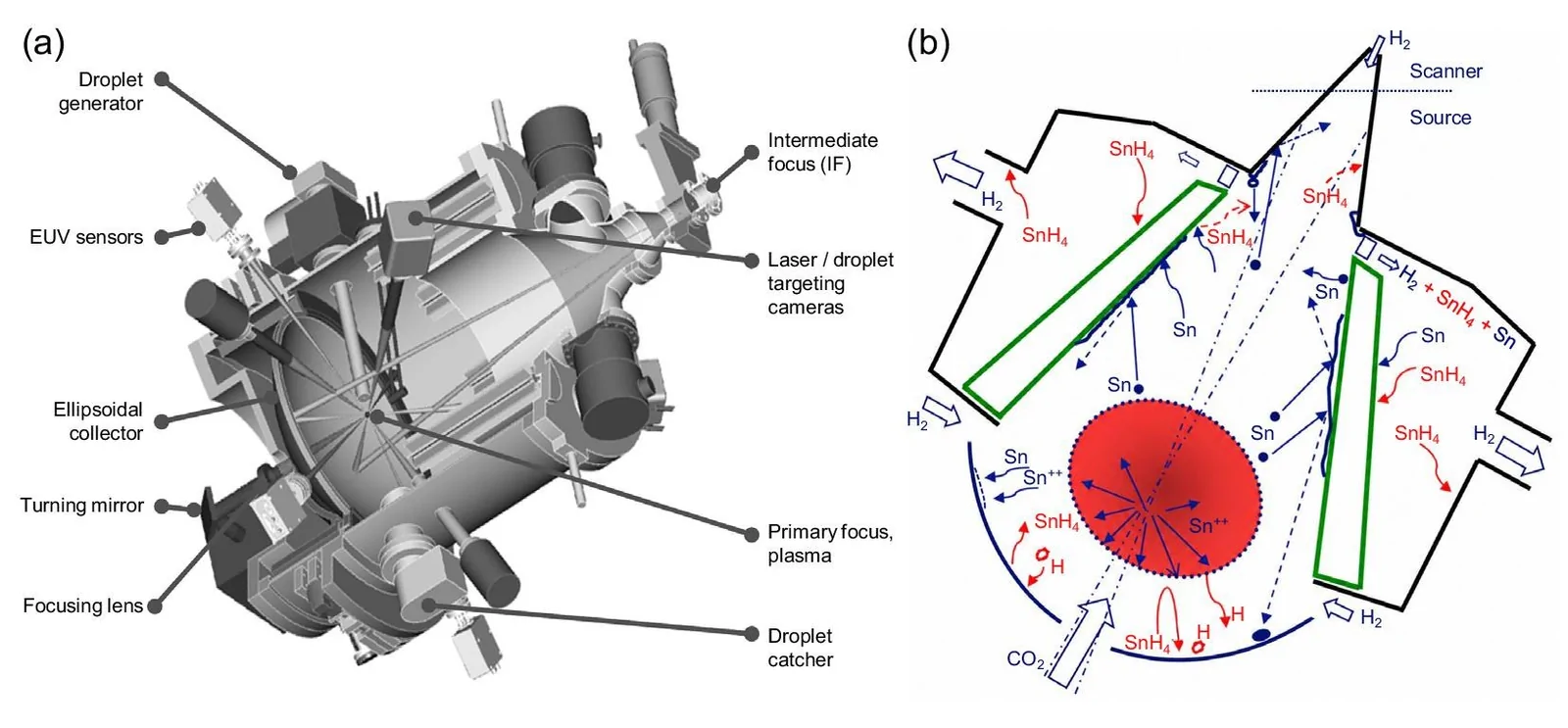
EUV light vessel consist of injection of buffer gas into the vacuum chamber. (a) The complicated components in EUV light vessel; (b) the simplified diagram showing the internal flow setup which involved the multiple inlets and outlets. The complicated flow problem can be simplified into several interacting jet flows in low pressure environment and their interaction with the debris. Panel (a) adapted from Ref. [138]; (b) adapted from Ref. [139].

A promising strategy for debris mitigation involves employing a hydrogen buffer gas [72], where supersonic gas flow establishes a Dynamic Gas Lock (DGL), facilitated by multiple and sometimes colliding gas inlets (see Fig. 7). Note that the use of a DGL is the mitigation approach currently adopted by ASML [2]. A couple of studies on DGL mechanisms highlight their effectiveness in reducing contamination and changing the properties of tin debris in EUV lithography systems. As mentioned before, the buffer H$_2$ gas can decrease the charge state of tin ions during microscopic atomic collision [124,125]. Actually, Ruzic group from UIUC [131] and Abramenko *et al.* with the collaboration with ASML [132] have performed the stopping cross-sectional measurement. It provides H$_2$ stopping cross-section for Sn ions in the wide energy range. In the meantime, the macroscopic stopping power and mitigation efficacy of the H$_2$ buffer gas have been extensively quantified. The macroscopic mitigation efficacy of the H$_2$ buffer gas has been extensively quantified through experimental campaigns by Ruzic and co-workers [133–135], which measured the energy dissipation of tin debris. In addition, Sun *et al.* [136] provided a theoretical analysis of the suppression ratio, elucidating how DGL parameters influence debris mitigation. Hao *et al.* [137] demonstrated practical implementation of DGL in cleanrooms, showing significant reductions in contamination levels.

The key component in DGL is the colliding jet, where from experimental point of view the complex DGL problem can be simplified into jet flow injecting into the low pressure environment (as depicted in Fig. 7). During the collision of the two jets, there is intense energy and momentum exchange, making this flow behavior highly valuable for applications in the lithography chamber [8]. Therefore, understanding the evolution of the buffer gas colliding jet structure, analyzing factors influencing this jet behavior, and revealing its mechanisms of heat and particle transport is important. As early as 1996, Kubiak *et al.* [140] designed a method for introducing a supersonic buffer gas into the EUV light source chamber to prevent deposition of metal target debris on the mirror surface. Compared to setups without the buffer gas, they found that the buffer gas increased the mirror’s lifespan by 105 times. Harilal *et al.* [104] used hydrogen, helium, and argon gases in the chamber to effectively clear tin debris and explored the use of external magnetic fields to slow down tin plasma propagation. Their results indicated that combining buffer gas with a magnetic field significantly reduced tin debris contamination without exceeding the EUV gas absorption limits. Wu *et al.* [141] applied visualization imaging systems and optical emission spectroscopy to quantify the mitigation capacity of hydrogen, helium, and argon buffer gases on laser-produced tin plasma plumes, discovering that at specific pressures, argon was more effective than hydrogen or helium in slowing high-energy tin ions. Sun *et al.* [142] studied the movement of tin debris in a light source chamber with argon and helium buffer gases and examined the effective range of different buffer gas pressures on tin ion debris mitigation. In recent experimental study, Chen *et al.* [143] examine the subsonic pipe jet expanding into near vacuum ambient which minics the light vessel environment. Their study examines how inlet flow rate, ambient pressure, and jet diameter influence the jet’s centerline velocity.

### Numerical approaches on assessing mitigation efficiency

To evaluate the effectiveness of these mitigation approaches, it is essential to develop a numerical method capable of capturing debris transport within a multiphysics environment. For example, Meng *et al.* [3] used numerical simulation for studying the transport of debris in EUV vessels under the assumption that the debris is dilute and small-sized. Liu *et al.* [144] examined numerically how debris particles transport and deposit mechanisms with multiphase explosion coupling with buffer gas stream.

In the case of EUV laser-induced plasma, debris particles and droplets are transported within a low-pressure hydrogen environment (implementing buffer gas with pressure of about 100 Pa [2,145]). The flow is compressible and features shock waves generated by both the supersonic buffer gas and the expanding hot plasma core. To model the motion of charged debris under these conditions of low pressure and high flow velocity, a common approach is the Euler–Lagrangian approach, where the flow is simulated via Eulerian way and the motion of debris is via the Lagrangian way by treating the debris as point particle. Note that this approach inherently neglects the early-stage of debris formation where particle–particle interactions and the finite-size effect may be significant [146]. In community of multiphase in fluid mechanics, the motion of particle can be well-described by Tsai–Maxey–Riley equation. It is originally derived by Gatignol, Tsai, Maxey, and Riley, describes the motion of small, rigid, spherical particles in a fluid. However, its application to debris transport in EUV plasma requires extensions to account for shock-driven high-speed flows, compressibility, rarefaction effects, and magnetic forces acting on charged particles.

The equation of motion for a small spherical particle in an incompressible flow considers the terms regarding fluid acceleration, Stokes drag $F_{\rm D}$, buoyancy $F_{\rm D}$, added mass $F_{\rm add}$ and Basset history force $F_{\rm Basset}$. For EUV debris, the traditional treatment is inadequate due to the conditions of high flow speed, strong effect of rarefaction, compressibility and the existence of magnetic force. For such the problem, there are two relevant parameters: particle Knudsen number $\mathrm{\it Kn_{\rm p}}$ (mean free path of background molecules over characteristic diameter of the debris particle) which quantities the degree of rarefaction from the perspective of the debris particle; and the particle Mach number $\mathrm{\it Ma_{\rm p}}$ (speed of the debris particle over the speed of sound) which defines degree of compressibility. It is noteworthy that although the Knudsen number defined by the system size is small at 100 Pa environment, however, the particle Knudsen number as defined by the particle size is large. It means that the rarefied effect can not be neglected at the scale of the particle.

In the condition of EUV vessel, $\mathrm{\it Kn_{\rm p}}$ and $\mathrm{\it Ma_{\rm p}}$ might be sufficiently large such that corrections are needed for the force terms in particle equation of motion. Parmar *et al.* [147] suggested a generalized drag force and compressibility corrections in the case of high-speed shock flows in rarefied environement. First, there are corrections for history force and added mass terms because of the compressibility effect. The Basset history force accounts for the effect of past particle acceleration on its current motion. For compressible high-speed flow, the history force is modified due to shock interactions and rarefied effects. For details of corrections for $F_{\rm add}$ and $F_{\rm Basset}$, we refer the reader to recent review on particle in high speed flow [148].

Moreover, it is important to note that the classical Stokes drag is no longer applicable in this regime and must be replaced by a non-Stokesian drag model, where the drag coefficient $C_{\rm D}$ depends intricately on both the particle Mach number ${Ma_{\rm p}}$ and the Knudsen number $\mathrm{\it Kn_{\rm p}}$. The seminal work by Loth *et al.* [149] provides a comprehensive summary of how compressibility and rarefaction effects alter $C_{\rm D}$ for a spherical particle.

Finally, regarding the background flow under low pressure, one might need to consider the chamber Knudsen number ${Kn_{\rm c}}$ (defined by the mean free path over the chamber size). There are basically four regimes depending on $\mathit{Kn_{\rm c}}$, which are continuum regime, slip regime, transition regime, and free molecular regime. For the main chamber, considering a characteristic length of 1 m and hydrogen buffer gas at a working pressure of 100 Pa, the Knudsen number $\mathit{Kn_{\rm c}}$ is less than $10^{-3}$ [3], indicating that the flow is well within the continuum regime and can be modeled using the Navier–Stokes equations. However, for components with small dimensions, such as the nozzle, the relevant length scale is reduced significantly, and one must account for rarefied flow conditions using approaches such as Direct simulation Monte Carlo, unified stochestic particle, Grad’s 13 moment equation, discrete unified gas kinetic scheme, general synthetic iterative scheme or some hybrid approaches, etc [150–156].

### Experimental technique on measuring flow at low pressure environment

**Figure 8.**
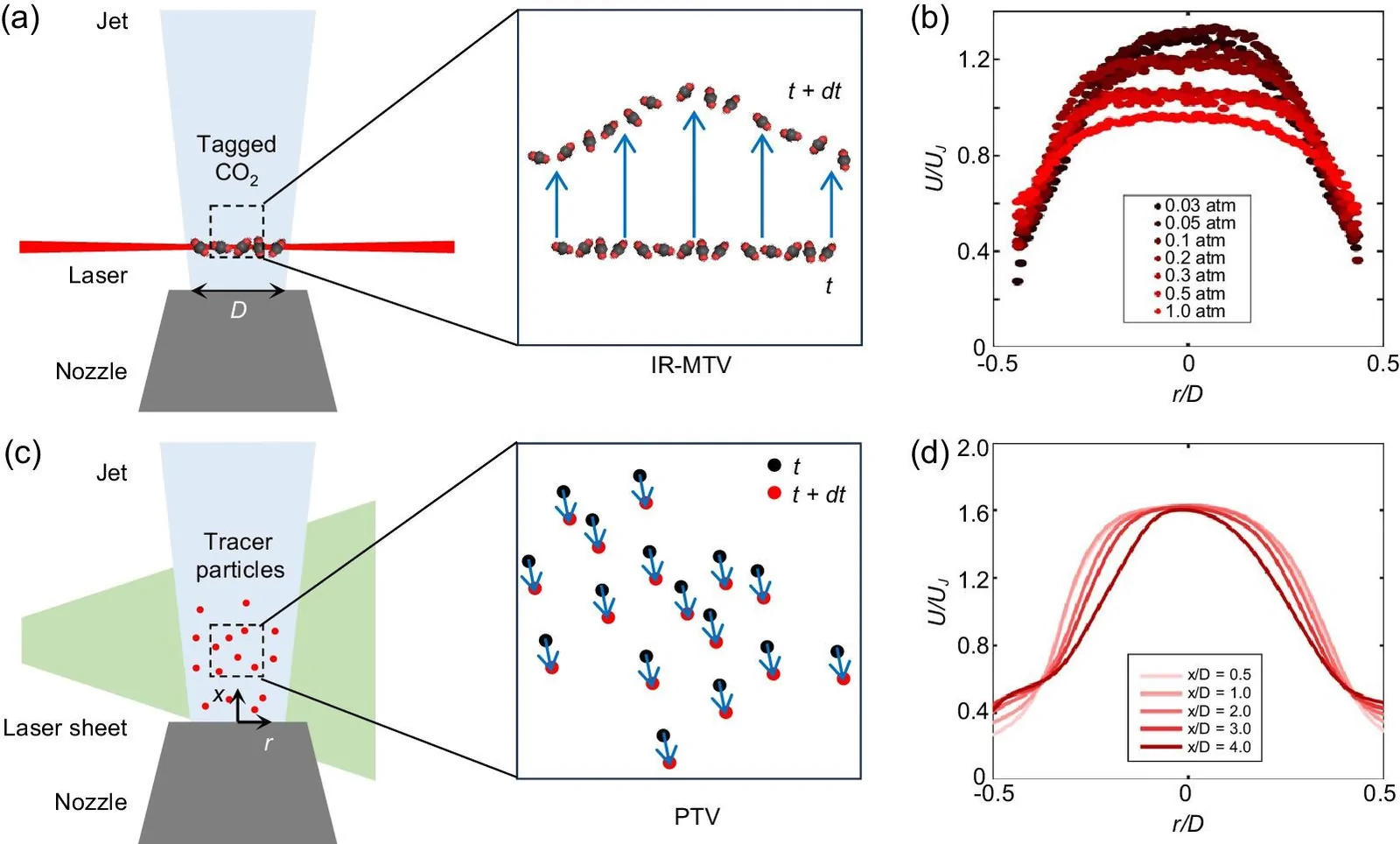
Two techniques for the flow measurement in low pressure environemnt using (a and b) infrared molecular tagging velocimetry; (c and d) particle tracking velocimetry. (b and d) show the radial distribution of axial velocity of the gas jet. Panel (b) adapted from Ref. [157]; (d) adapted from Ref. [143].

To conduct comprehensive study on how the jet and debris behave in low pressure environment, it is essential to develop the flow measurement under such condition. Debris in EUV lithography is mitigated by buffer-gas jets in near-vacuum vessels at ambient pressures of tens of pascals. Quantitative measurements of the jet flow under these conditions are difficult and remain scarce. In a recent study spanning back pressures from 3000 Pa to atmospheric levels (101325 Pa), Song *et al.* [157] used infrared molecular tagging velocimetry (IR MTV) to examine CO$_2/$N$_2$ microjets (Fig. 8a). They observed that as the pressure decreases, the initially uniform “top-hat” velocity profile gradually transitions to a symmetric Gaussian-like distribution, accompanied by a centerline velocity that exceeds the mean exit velocity, as shown in Fig. 8b. At lower pressure levels, Huffman and Elliott [158] investigated supersonic, rarefied, axisymmetric jets using particle image velocimetry (PIV) and molecular tagging velocimetry (MTV) at pressures of 500–1000 Pa. They found that tracers of 100 nm and 1.0 *μ*m could both recover mean velocities with errors of less than 5% and 15%, respectively, and that PIV could successfully resolve shock positions. Park *et al.* [159] extended the PIV and MTV measurements to 133 Pa, identifying a supersonic laminar regime featuring a single-barrel shock in the near field and, downstream of the Mach disk, a long annular viscous layer that sustains an extended potential core. Most recently, Chen *et al.* [143] introduced a dedicated near-vacuum facility in which the jet experiments can be conducted at an ambient pressure of 13.9 Pa and flow rates as low as 0.28 SLPM (see Fig. 8c and d). By employing particle tracking velocimetry (PTV), they revealed an extended potential core and persistent “top-hat” velocity profiles, which are consistent with the supersonic laminar regime observed by Park *et al.* [159], despite the ambient pressure being one order of magnitude lower.

## CONCLUSIONS AND PERSPECTIVES

In this review, we summarize the key flow physics involved in the prepulse, main pulse, and debris transport/mitigation stages of LPP-EUV sources. The laser–plasma interaction, tin droplet dynamics, and debris transport under rarefied conditions play critical roles in addressing the two major challenges in EUV lithography: conversion efficiency and debris contamination. This field continues to grow rapidly, representing an exciting and emerging area within fluid mechanics.

Over the past decades, ASML NXE series for LPP-EUV lithography has achieved significant power increase from 10 W in 2010 (NXE:3100) to 300 W in 2024 (NXE:3800E) while simultaneously improving conversion efficiency and debris mitigation. These advances have been tightly connected to progress in fluid mechanics: precise tin droplet shaping through long prepulses, optimized plasma–droplet interaction, and high-speed hydrogen flow control for debris suppression. Milestone implementations include the introduction of prepulse technology in NXE:3300B, MOPA-assisted droplet deformation for higher CE, and continuous refinement of dynamic gas lock systems. In future, ASML and partners are actively developing a three-pulse strategy—droplet breakup, sheet expansion, and final plasma generation—to push CE and support future 1 kW-class EUV power systems. Many gaps needed to be filled in the future generation of EUV lithography system, where the outlook for the key flow physics regarding the improvement of conversion efficiency and debris mitigation in the future are summarized below:

• Motivated by the potential for deeper penetration of shorter-wavelength lasers into plasma and consequently higher energy deposition in the target, solid-state lasers (for example, Nd:YAG) are being explored as candidates for next-generation devices, particularly as prepulses [8,39,40]. Detailed numerical studies have examined wavelengths ranging from 1 to 10 *μ*m [38,39]. These studies demonstrated a strong dependence of laser absorptivity and conversion efficiency (CE) on wavelength [39]. Specifically, increasing the pulse duration of a 2 *μ*m laser was found to enhance CE [38], and simulations further showed that a 2 *μ*m laser yields higher CE than a 1 *μ*m when irradiating a solid planar tin target [60].

• Next generation of LPP-EUV lithography adopts three-pulse strategy. It is a future trend to examine how multiple prepulse being arranged for optimized plasma-droplet interaction. In this problem, simulations are ideally suited for optimizing performance through laser pulse shaping (for example, multi-pulse sequences) and droplet size adjustments [39].

• The development of synchronized measurement systems combining laser spectroscopy and radiation imaging for Sn plasmas represents a critical direction for future experimental diagnostics [8,157,160]. Such integrated systems can simultaneously capture the spectral signatures of plasma species and the spatial-temporal evolution of droplet deformation, enabling direct correlation between plasma conditions (electron density, temperature, and ionization states) and hydrodynamic responses. This approach will provide unprecedented insights into the energy transfer mechanisms from laser to plasma and subsequently to the droplet, particularly during the critical nanosecond-to-microsecond transitions where plasma expansion and droplet deformation are strongly coupled.

• The prevailing numerical studies on debris transport often rely on point-particle assumption. However, during the early stage of debris formation, dense matter has to be considered instead of dilute and small-sized debris. In high-speed, rarefied flows, non-continuum effects and finite particle size can drastically alter drag and heating. Furthermore, when debris is charged and subject to electromagnetic fields, its behavior becomes coupled to the plasma, moving beyond simple Lagrangian tracking. Therefore, the future of accurate simulation lies in developing hybrid or fully coupled methods that can transcend the point-particle approximation, directly resolving the intricate, two-way interactions between the debris and the extreme multiphysics environment it inhabits.

• To overcome the computational cost and spatiotemporal challenges of multiscale multiphysics coupling, future strategies may integrate AI with fluid dynamics and plasma physics (“AI for Science”). Traditional approaches are often limited by disparate time-step constraints. Emerging data-driven methods, such as Physics-Informed Neural Networks (PINNs) and operator learning (for example, DeepONets), offer a promising solution by replacing expensive PDE solves with pre-trained surrogate models [161]. Trained on high-fidelity simulations, these models can rapidly predict complex multiphysics behavior, including EOS, radiation transport properties.
